# ‘Invisible actors’—How poor methodology reporting compromises mouse models of oncology: A cross-sectional survey

**DOI:** 10.1371/journal.pone.0274738

**Published:** 2022-10-20

**Authors:** Elizabeth A. Nunamaker, Penny S. Reynolds

**Affiliations:** 1 Animal Care Services, University of Florida, Gainesville, Florida, United States of America; 2 Department of Anesthesiology, Statistics in Anesthesiology Research (STAR) Core, College of Medicine, University of Florida, Gainesville, Florida, United States of America; Turun Yliopisto, FINLAND

## Abstract

The laboratory mouse is a key player in preclinical oncology research. However, emphasis of techniques reporting at the expense of critical animal-related detail compromises research integrity, animal welfare, and, ultimately, the translation potential of mouse-based oncology models. To evaluate current reporting practices, we performed a cross-sectional survey of 400 preclinical oncology studies using mouse solid-tumour models. Articles published in 2020 were selected from 20 journals that specifically endorsed the ARRIVE (Animal Research: Reporting of In Vivo Experiments) preclinical reporting guidelines. We assessed reporting compliance for 22 items in five domains: ethical oversight assurance, animal signalment, husbandry, welfare, and euthanasia. Data were analysed using hierarchical generalised random-intercept models, clustered on journal. Overall, reporting of animal-related items was poor. Median compliance over all categories was 23%. There was little or no association between extent of reporting compliance and journal or journal impact factor. Age, sex, and source were reported most frequently, but verifiable strain information was reported for <10% of studies. Animal husbandry, housing environment, and welfare items were reported by <5% of studies. Fewer than one in four studies reported analgesia use, humane endpoints, or an identifiable method of euthanasia. Of concern was the poor documentation of ethical oversight information. Fewer than one in four provided verifiable approval information, and almost one in ten reported no information, or information that was demonstrably false. Mice are the “invisible actors” in preclinical oncology research. In spite of widespread endorsement of reporting guidelines, adherence to reporting guidelines on the part of authors is poor and journals fail to enforce guideline reporting standards. In particular, the inadequate reporting of key animal-related items severely restricts the utility and translation potential of mouse models, and results in research waste. Both investigators and journals have the ethical responsibility to ensure animals are not wasted in uninformative research.

## Introduction

The laboratory mouse is a well-established and common research model used to study human diseases, and a key link in the translation of bench experiments to clinical trials. In cancer research, mouse models are major players in three domains. First, mouse models enable insight into the genetic, mechanistic, and phenotypic mechanisms and interactions underlying the pathogenesis of cancer initiation and tumour formation. Second, they serve as *in vivo* platforms for drug discovery and therapeutic screening and evaluation. Finally, mouse models permit direct testing of the relationship of tumorigenesis to various environmental factors not possible in clinical studies of humans [1].

However, the ‘mouse model’ in oncology research is not a monolith. Mouse strains are varied, animal genotypes are manipulated, induction methods are wide-ranging (e.g. engraftment, syngeneic, orthotopic, and genetically-engineered models), and disparate methods of determining marker expression are used [1, 2]. While lab bench methods are usually well described and thoroughly documented in literature reports, the animals themselves have been ‘invisible actors’. Information on routine care, housing, welfare measures (such as anaesthesia, analgesia, euthanasia, [3]), and animal signalment (strain, sub-strain, age and sex) have all been documented to influence both progression of specific cancers and expression of experimental outcomes [4]. An additional consideration is that many cancer models are associated with high rates of lethality and potential for suffering, so reporting of care and welfare measures are necessary for assessing if studies do in fact meet basic ethical standards. However, this information has not been prioritised in much of the literature. The omission of animal-related details, intentional or not, may be due in part to the perception of mice as disposable, inter-changeable commodities, or “furry test tubes” [5]. Without complete and accurate description of all methods related to the specific animal model, including care and welfare, it will not be possible to assess the relevance of the models, interpret and generalize results, or even determine if the research followed best-practice scientific and ethical standards.

We performed a cross-sectional survey [6] of studies of solid-tumour oncology mouse models to evaluate the reporting of items specifically related to animal care and welfare, animal-related cancer aetiology, and endpoint expression. We confined searches to recent major cancer journals that explicitly endorsed the ARRIVE (Animal Research Reporting: In Vivo Experiments) reporting guidelines [7, 8] because these guidelines provide an objective benchmark for quality reporting expectations [9]. The primary intent of this survey was neither to synthesize evidence (as with systematic reviews), nor perform a complete evaluation of adherence to all reporting items identified by ARRIVE. Instead, the main objective of this investigation was to provide a prevalence snapshot of commonly-overlooked reproducibility ‘risk factors’ specifically associated with animal use, humane care, and welfare. To ensure rigorous review, we followed standards for conduct and reporting of scoping reviews (The PRISMA Extension for Scoping Reviews (PRISMA-ScR) [10]. Oncology studies utilizing mouse models were evaluated for reporting of items in five key animal-specific domains (ethical oversight assurance, animal signalment, husbandry, welfare, and euthanasia), and evaluated for the extent of reporting compliance and major gaps. We also evaluated reporting of simple study validity items (sample size, sample size justification, and bias minimisation) to enable comparison with other, more general, reviews. We discuss how reporting gaps identified in this survey limit the utility and translatability of mouse oncology models, and provide a list of targeted recommendations to improve the quality of these studies.

## Materials & methods

### Eligibility and screening

**Fig 1:**
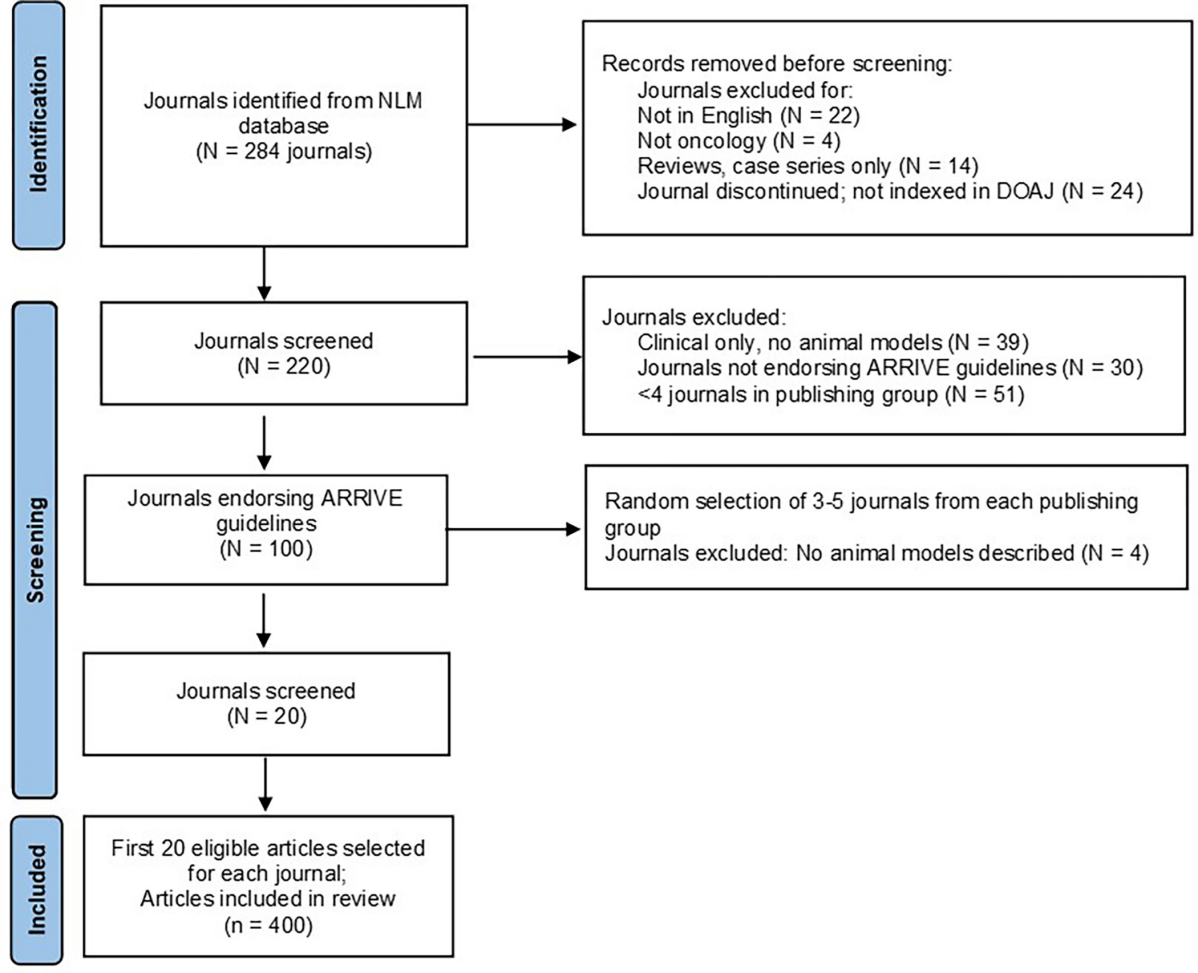
Flow diagram for journal identification, article selection, screening, inclusion, and exclusion.

Data were extracted from 400 articles in 20 oncology research journals representing six publishing groups (**Fig 1**). The study was purposefully restricted to a single year (2020). A total of 284 journals related to oncology research were identified and screened. Journals were selected if the primary focus was on preclinical studies involving animal use, and if they explicitly endorsed ARRIVE reporting guidelines in the Instructions to Authors [7, 8]. Journals were excluded if subject matter was predominantly or exclusively clinical and/or molecular, as indicated by both electronic search on the terms ((mouse OR mice OR murine) OR preclinical OR animal) and visual search of article titles, abstracts, and main text in each journal. Impact factors ranged from 2.97 to 26.5, as determined from the 2020 Journal Citation Reports (Clarivate Web of Science). We selected the first 20 articles in each journal for the year 2020 that described original experimental research involving mouse oncology models. Clinical or epidemiological studies, *in vitro* studies, letters to the editor, conference abstracts, and reviews were not included. Journal and article selection processes are described in more detail in the **S1 File: Supplementary Methods.**

### Data

Both authors independently screened each article by examining Materials and Methods, Results, and article supplementary files (if provided) for reference to experiments involving mice. Key reporting items in five domains (ethical oversight assurance, animal signalment, animal husbandry, welfare-related items, and euthanasia), identified from the relevant sections of ARRIVE guidelines [7, 8] and itemised in a checklist (**S1 File: Supplementary Methods**) were scored as reported (1) or not reported (0). Only information included in the main text and supplementary methods was included. Information reported in figure legends but not mentioned elsewhere in the text was not considered as reported. The subset of articles that reported measurements of subcutaneous tumour volume as an experimental outcome (n = 290) were scored for tumour burden metrics reporting (method, sites, volume, maximum tumour size, time to maximum tumour size). All articles were scored for simple study validity metrics (total sample size, sample size justification, randomisation, blinding; **S1 File: Supplementary Methods**).

Both authors scored all articles individually in separate spreadsheets (Microsoft Excel 2019; Microsoft Corporation, Redmond, WA). Discrepancies between scored items were flagged electronically. Discrepant entries were then compared to information in the original article for correction if necessary, and remaining ambiguities or divergence resolved by consensus. Data were imported into SAS 9.4 (Windows 10PRO x64; SAS Institute Inc., Cary, NC) for analysis. Further details are given in **S1 File: Supplemental Methods.**

### Statistical analyses

This was a descriptive study rather than a hypothesis-testing study. Data were itemized and summarized by counts and percentages. Patterns of reporting compliance for care and welfare items were analysed using two-level hierarchical generalised random-intercept models, with articles clustered within journal, no predictors, and dichotomous (binary) outcomes [11, 12]. There were no previously published estimates for expected between- and within-cluster variances. Therefore, sample sizes for journals and articles per journal were selected to give reasonable estimates and precision for model parameters [13]. Simulations have indicated that confidence intervals with approximately correct coverage rates and minimal downwards bias can be obtained with approximately 20 observations per cluster and at least 20 clusters [14]. Poor reporting of study validity items precluded formal analysis.

The binary response (yes/no) for each reporting item was modelled as *η_ij_* = *γ*_0_+*u*_0*j*_ where *η_ij_* is the log odds of a given item being reported for article *i* in journal *j*, *γ*_0_ is the random intercept component representing the log odds of an item being reported for a given journal, and *u*_0*j*_ is the journal-level error, with *u*_0*j*_ assumed to be normally distributed with mean 0 and variance τ_0_. The probability of reporting compliance for each item was calculated as $\phi_{\mathrm{ij}}=e^{\eta_{\mathrm{ij}}}/(1+e^{\eta_{\mathrm{ij}}})$. The amount of variation in item reporting accounted for by journal membership was assessed by intraclass correlation coefficients (ICC). ICC is estimated as the proportion of variance that can be attributed to between-journal variation: ${ICC=\sigma }_{jour}^{2}/(\sigma_{jour}^{2}+\sigma_{e}^{2})$, where $\sigma_{jour}^{2}$ is level-2 or between-journal variation, estimated from the covariance parameter estimate, and $\sigma_{e}^{2}$ is the level-1 or article-level variance, estimated as *π*^2^/3 for the standard logistic distribution. (The variance for a hierarchical generalized linear model with binary outcomes is directly determined by the population mean [11, 12]). The ICC can also be interpreted as the correlation ρ of the response for any two articles selected at random from the same journal [15]. Models were fitted using Laplace estimation in SAS *proc glimmix* (SAS v.9.4, SAS Institute, Cary NC; **S1 File: Supplemental Methods;** [11]).

## Results

### General

**Fig 2:**
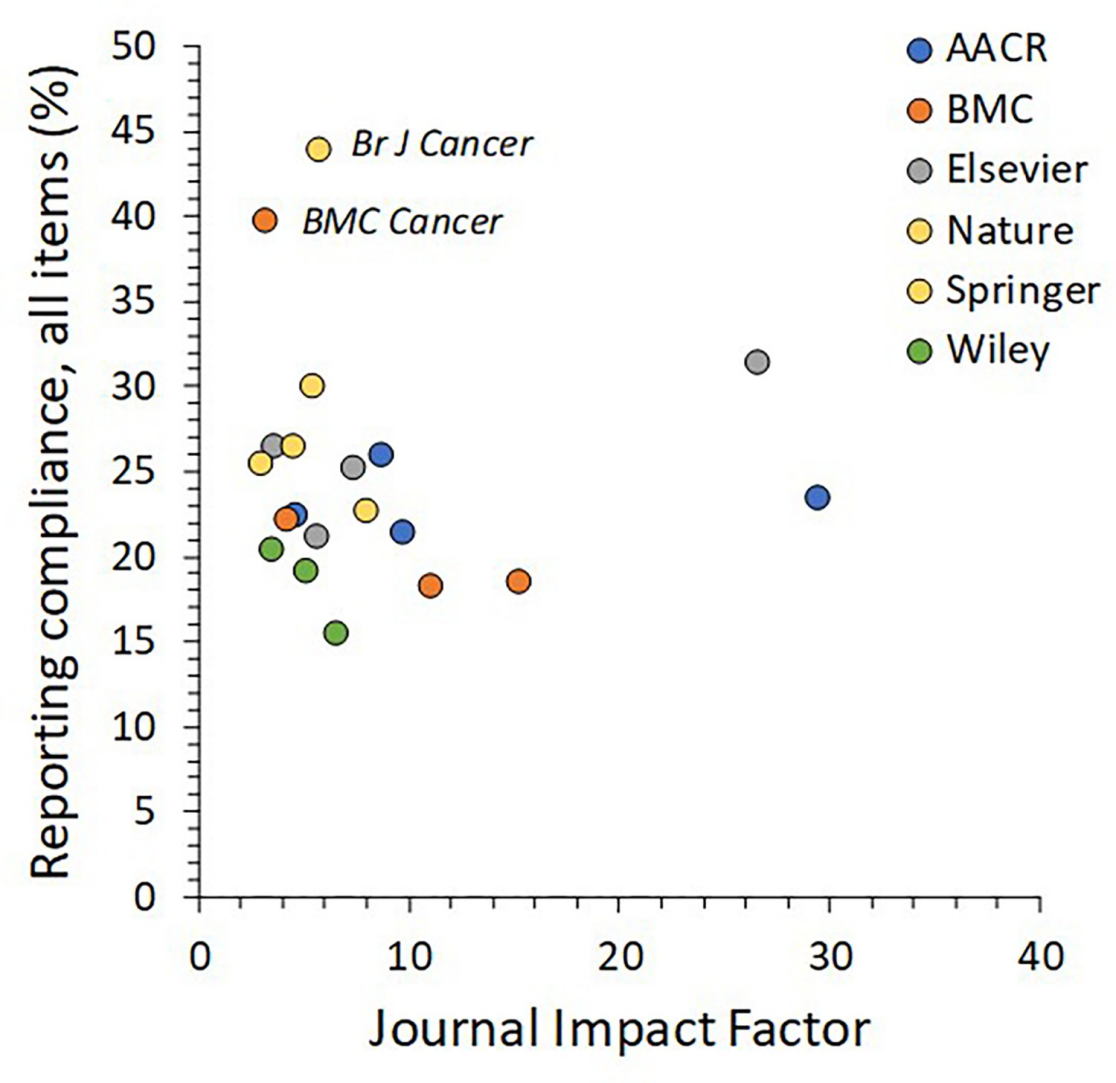
Association of journal impact factors and percent overall animal care & welfare reporting for mouse-based oncology studies in 20 journals.

The summary of reporting compliance for care and welfare items is given in **Table 1.** Count summaries by journal are given in the **S2 File: Supplemental Results**. Median reporting compliance was 23% (IQR 21, 27%) for all reporting items across journals in this survey. There was no apparent relationship between percent compliance and journal or journal impact factor (**Fig 2**). Unadjusted correlation for care and welfare items with journal impact factor was r = −0.05 (95% confidence interval −0.48, 0.40), and the correlation of validity items with impact factor was r = −0.18 (95% confidence interval −0.57, 0.29).

**Table 1:** Summary of reporting probability *ϕ* (proportion of articles reporting a given item, corrected for journal membership) with 95% confidence intervals, and intraclass correlation (ICC*)*, estimated from two-level hierarchical models of articles nested within journal.

			Reporting Probability			
		Number of articles, *n*	*ϕ*	95% CI		ICC
***Ethical oversight***	Institutional approval	331	0.838	0.779	0.883	0.28
	Approval number	105	0.236	0.160	0.332	0.38
	Care and use guidelines	174	0.430	0.350	0.515	0.29
***Signalment***	Nominal strain identifiers	356	0.909	0.851	0.945	0.35
	Verifiable strain identifiers	34	0.045	0.018	0.106	0.64
	Source or derivation	290	0.745	0.659	0.816	0.33
	Age	325	0.813	0.766	0.853	0.24
	Sex	287	0.725	0.659	0.782	0.27
	Weight	45	0.082	0.043	0.149	0.45
***Husbandry***	Caging	16	0.028	0.011	0.071	0.41
	Number of mice per cage	14	0.018	0.005	0.058	0.59
	Enrichment	4	0.000	0.000	0.056	0.00
	Temperature	28	0.033	0.011	0.093	0.71
	Photoperiod	45	0.072	0.034	0.143	0.55
	Food and water access	47	0.096	0.058	0.156	0.36
	Feed type	16	0.021	0.006	0.066	0.60
	Acclimation	18	0.044	0.023	0.083	0.25
***Welfare***	Anaesthesia	59	0.127	0.082	0.192	0.33
	Analgesia	11	0.022	0.008	0.059	0.34
	Humane endpoints	65	0.163	0.129	0.202	0.00
***Euthanasia***	Method identified	78	0.135	0.066	0.255	0.72
	Not specified	213	0.530	0.419	0.637	0.37
	Not reported	108	0.261	0.202	0.329	0.27

Reporting (**Table 1**) was highest for minimal ethical oversight statements (84%), nominal strain identification (91%) and animal age class (81%), and lowest for animal husbandry (0–10%), welfare (2–16%), and euthanasia (7%) items. The intraclass correlation coefficients (ICC) indicate how much of the total variation in the probability of reporting a specific item is accounted for by journal. ICCs were for the most part moderate to poor, reflecting rates of reporting that were either uniformly high (e.g. ethical oversight items) or uniformly poor (e.g. welfare items) across all journals. Items with ICC of zero reflect either almost no reporting at all (enrichment, n = 4/400 articles), or rare intermittent and inconsistent reporting across multiple journals (humane endpoints n = 65/400). Items with the highest ICC resulted from low overall frequency of reporting, resulting from high reporting concentrated in only one or two journals and poor reporting for all other journals. For example, ‘temperature’ has an overall reporting frequency of 3.3% (ϕ = 0.033) but an ICC of 0.71, reflecting 13 reports located in only two journals, and 15 reports in the remaining 18 journals (**S2 File: Supplemental Results**).

### Ethical oversight

Approximately 84% (*ϕ* = 0.838) of articles explicitly reported that prior institutional approval was obtained, and 43% reported adherence to recognised and verifiable standards of humane animal care and use. However, fewer than one in four (24%) provided verifiable institutional protocol or license numbers, and 26/400 (7%) did not report any verifiable declaration of either institutional approval or care and use guidelines. One article claimed that their study “was not required to complete an ethical assessment”, and one study stated that approval had been obtained “retrospectively”. Approval numbers and oversight statements of six articles were identical to those reported in articles on different topics published in different journals by unrelated research groups. Animal Welfare Assurance Numbers, which do not refer to individual animal use protocols but are granted to Public Health Service (PHS) awardee institutions, were reported by seven articles.

### Animal signalment

Signalment is the complete description of the animal model itself, and should include unambiguous identification of strain, strain source (a recognised vendor, repository, or laboratory), age, sex, and if possible, body weight, as stipulated in the ARRIVE guidelines. For genetically modified strains, either reference to recognised official consensus identifiers (RRID, vendor stock or strain numbers), or a complete description of genotype derivation should be reported [16]. Although 91% of surveyed articles provided at least a nominal strain identification, only 75% provided a source or recognised vendor, and fewer than 5% of articles provided clear, complete and verifiable genotype identifiers or a sufficiently detailed description of breeding stock development. In 9% (44/400) of surveyed studies, strains used were either not identified, or only vague descriptors (such as “nude mice”) were provided. The journal *Cancer Cell*, which uses a structured reporting format for methods (STAR-Methods, [17]) had the most thorough documentation of strain identifiers, with 12/20 articles supplying complete information.

The majority of research articles reported sex (73%) and age (81%). Nearly half (47%) reported using animals 4 to 6 weeks of age, although it was not clear if these were the ages at which animals were acquired, or if they were the ages at which experimental manipulations occurred. Body weights were reported for only 45/400 (11%) of surveyed studies.

### Husbandry

Key husbandry information was poorly reported. Basic information on caging, cage density (number of mice per cage), and enrichment was almost never described (0–3%). Environmental variables temperature and photoperiod were also poorly reported (3% and 7% respectively).

### Welfare

Descriptions of welfare-related assessments and procedures (analgesia and anaesthesia, post-operative and palliative care, and humane endpoints) were poorly reported. Anaesthesia and analgesia use were reported by 13% and 2% of articles respectively. Articles reporting use of specific agents rarely provided necessary information on methods of administration, dose, route, concentration, manufacturer, indications for use, and/or administration schedules. Less than 1% (3/400) papers described pre-emptive analgesia use, 2.5% (10/400) reported using post-operative analgesia, and 2% (8/400) reported use of opioids. Most studies reported monitoring experimental animals for days to weeks post-tumour induction and before euthanasia, and 52% reported some sort of “survival analysis” using methods for time to event data (e.g. Kaplan-Meier estimates, Cox proportional hazards regression). However, only 16% (65/400 studies) reported specific humane endpoints. No study reported direct assessments of pain-related behaviours or response to palliative care measures.

Tumour volume is commonly used to assess disease progression, tumorigenicity, and response to therapeutic intervention [18], and tumour burden is a critical humane endpoint [3]. Specific methodological details for tumours are not explicitly identified in the ARRIVE guidelines. Nevertheless, it should be apparent that all methods used to determine a key experimental endpoint should be reported in adequate detail, and specific guidelines for reporting tumour burden and humane endpoints have been available for over a decade [3]. Of the 290 articles describing subcutaneous tumour volume as endpoint, pertinent descriptors necessary for volume determination were not reported by the majority of papers. Twenty-seven percent (78/290) did not report the anatomical site of tumour induction or gave only a vague description, 52% (152/290) did not report whether induction sites were unilateral or bilateral, 53% (152/290) did not report the measurement tool used (e.g. callipers), 29% (85/290) did not report the volume calculation formula, 72% (210/290) did not report the *a priori* maximum allowable tumour size for humane endpoint, and 59% (172/290) did not report the *a priori* maximum duration allowable for tumour growth prior to euthanasia. Inspection of results and figures suggested that at least 39% of reported studies (114/290) allowed tumours exceed the recommended limit of 1500 mm^3^ without scientific justification [3], 9% (46/290) showed animals with tumours exceeding 3000 mm^3^, and 8 articles reported tumour sizes exceeding 6000 mm^3^. Another 16% (46/290) either did not report volumes at all (although describing the methods of doing so), or reported only ‘relative’ or ‘normalised’ volumes or nonstandard metrics which could not be assessed. There was also considerable variety in methods of calculating tumour volume. Nearly half (49%, 141/290) of surveyed studies measured tumour size with external callipers and calculated volume as a quadrangular prism in two dimensions (length x width^2^/2). However, the remainder described use of up to 11 different formulae or variations. No paper reported determination of associated measurement errors or intra-observer variation.

### Euthanasia

Euthanasia methods were explicitly identified in only 14% of surveyed articles. Only two journals (*BMC Cancer*, *British Journal of Cancer)* consistently reported euthanasia methods (90%; 36/40). Although over half (53%) all surveyed articles reported that animals were euthanized, no methods were identified or described in these papers. The remaining 27% of articles did not report any method of animal disposition before tissue harvest.

### Study validity

Although all studies reported results of null hypothesis statistical tests, few studies reported verifiable information for total numbers of animals used, formal sample size justification, or bias minimisation methods (randomisation, blinding). Only 15% (59/400) articles provided ‘total’ sample sizes, and 31% (124/400) gave a sample size per intervention arm. However, these numbers are likely an under-estimate of the number of animals used, as animal loss due to attrition and discarded experiments was not recorded, nor was it clear how many whole-animal experiments were actually conducted, or even how many intervention arms were tested. Formal sample size justification using power calculations was claimed by 7 papers (2%), although descriptions were too incomplete to allow verification. Another 16 papers provided other unverifiable forms of sample size ‘justification’, based on ‘previous experience’, ‘mouse availability and feasibility’, numbers ‘as small as possible to produce valid results’ or the ‘number used to obtain statistically significant results’. ‘Randomisation’ was claimed by 41% (165/400), with apparent stratification on tumour volume, animal weight, or age by 7 (<2%). Only 4 articles described using software or a random numbers table, and none described the randomisation method or the unit of randomisation. Two articles described as ‘random’ allocation that was sequential or alternating, respectively. Blinding was mentioned by 16 articles (4%), but none described how concealment was performed.

## Discussion

Mice are the ‘invisible actors’ in much pre-clinical oncology research. In spite of explicit endorsement of the ARRIVE reporting guidelines by all journals in this survey, reporting of animal-related information was inadequate. Descriptions of experimental techniques and procedures were emphasised at the expense of critical animal-related detail, and many details essential for assessing both study reliability and animal welfare were not reported. The widespread failure to report these details represent significant methodological omissions in preclinical oncology research.

Unfortunately, the results of this survey are consistent with those of other recent reviews that have found poor reporting compliance for other research specialties [19, 20]. There is increasing concern that the lack of reproducibility of much animal-based research is directly related to poor methodological documentation of critical information both ‘inherent to the animals’ (such as strain, age, sex etc.), but also those ‘extrinsic factors of the animals’ environment … that systematically influence the experimental outcomes’ [21–23]. Without these details, much of the evidence claiming translation potential of mouse-based oncology models will be suspect. Complete and accurate reporting of experimental details is crucial for assessing model relevance, potential sources of variation and model disparity, translation potential, and (not least) if animal-based research has been conducted in compliance with best-practice animal welfare standards.

Ethical oversight of animal research is a fundamental research requirement. All journals in the current survey expressly stipulated that prospective studies required approval from the relevant institutional oversight committee before research animals were obtained or used. Therefore, it was both disappointing and unexpected that unambiguous and verifiable statements of institutional approval showed much less than 100% compliance, nearly one in ten studies failed to report any verifiable ethical oversight information at all, and some provided information that was demonstrably false. Poor animal care and use is poor science. Without verifiable ethical oversight information, it is impossible to tell if ethical review of the animal experiments was actually performed, if studies were conducted under appropriate ethical oversight, or if the work followed best practices for humane care and use. Plagiarised or false ethical oversight information is research misconduct.

Standardized, genetically defined mouse strains and stocks are primary biomedical research tools. However, in this survey, <10% of studies reported verifiable strain descriptions or used standardised nomenclature. This is of concern, because mice, and especially inbred strains, are subject to both obvious and quiet genetic mutations with each round of breeding [16, 24–26]. These mutations can be due to genetic drift with differential fixation, or genetic contamination resulting from breeding colony mismanagement [27]. Quiet mutations are the most problematic because they do not result in a readily visible phenotype, and can go undetected unless genetic stability testing is performed routinely [16]. Sub-strains of inbred lines produced by different commercial vendors, or continuous in-house breeding programs, may differ both genetically and phenotypically, thus contributing to variation and compromising data interpretation [28]. The scientific community must follow basic guidelines for breeding and describing research animals, such as those endorsed by FELASA (Federation of European Laboratory Animal Science Associations) [16]. Meticulous and accurate identification of mouse lines used is essential to ensure the assumed genetic model is in fact the correct model for purpose, facilitate scientific communication, and improve reproducibility. Without specific information on genetic background and strain derivation, it will not be possible to assess model relevance, identify appropriate controls, or interpret and generalize the results in a meaningful way [28, 29].

Descriptions of housing and husbandry practices are frequently overlooked in methods reporting, and in this survey were rarely reported. These omissions are of concern for study reproducibility in general, because housing and husbandry conditions can have profound effects on health and welfare of mice, and can be a cause of phenotypic variation. More specifically for oncology studies, housing and environmental conditions can greatly affect rates of tumour induction, invasion or remission, and response to test interventions. For example, both tumour formation and development in mice are affected by solitary versus social housing. Social isolation stress enhances tumour invasion, metastasis [30–34], tumour growth [34–44], and gene expression, and attenuates response to chemotherapy [35]. Compared to solitary animals, group-housed animals typically have smaller tumours and increased rates of tumour regression or rejection [40, 45, 46]. Additional environmental factors affecting tumour kinetics and gene expression include housing on ventilated racks [47–55], temperature (heat or cold) stress [56–62], bedding type [63], and enrichment [62, 64, 65]. Handling methods [47, 66] and the diet fed [67–72] also contribute to animal stress, and therefore may be expected to influence tumour growth and metastasis.

Results of this survey support prior observations [19, 73, 74] that preclinical research studies do not consistently report use of anaesthesia, analgesia, or other pain control measures. Even if surgery is not performed, general anaesthesia is generally used as a restraint agent in imaging studies. Choice of anaesthetic agents can introduce considerable experimental artefact and must be identified and justified [3]. Further, it is an ethical imperative to minimize pain and distress of animals used in invasive research. Pain management can be a methodological challenge if anaesthesia or analgesia agents have the potential to affect experimental endpoints, such as engraftment, tumour growth, or metastasis [75–77]. Thus, both cancer pain and methods of pain control have the potential to act as meaningful confounding factors [74]. Nevertheless, there is no good reason why responsible pain alleviation cannot be used [73]. Pain is a common clinical effect of many cancer types, and pain management is an integral part of human and veterinary oncology practice. Most cancer studies are conducted under the assumption that major morbidity and mortality will result from tumour progression without intervention. Analgesic use promotes welfare in animal oncology models by sustaining tumour growth to predetermined experimental endpoints without undue animal suffering [78]. Pre-emptive, perioperative and follow-up administration of pain relief measures are required for major and/or multiple survival surgeries, and studies with long post-injury monitoring periods. Analgesia should be the default for research protocols, and there should be very high scientific and ethical bar for withholding analgesia.

Given that many oncology studies have the potential for animal suffering and death, it is of major concern that information on welfare monitoring, humane endpoints, and euthanasia was reported so infrequently. Humane endpoints must be defined for so-called “survival studies” because death as an endpoint is discouraged by most reputable ethical oversight bodies. Failure to euthanize animals at predefined humane endpoints can lead to significant animal suffering and poor welfare. Potential adverse events and clinical signs associated with the therapeutic compounds under test should also be categorised *a priori*, identified and reported, especially if long-term study goals include translation.

Specification of humane endpoints should prioritise clear descriptions of methods for determining tumour burden, and predefined limits to maximum permissible tumour burden and duration of tumour growth. Because tumours vary in size and aggressiveness depending on the cancer type and location, ethical expectations are that protocols must also include clearly defined study-specific humane endpoint criteria, descriptions of monitoring frequency, and methods used to minimise suffering of the animals. More conservative tumour burden limits must also be considered if multiple tumours are present. Rigorous specification of these key metrics is necessary, both as reliable measures of tumorigenesis and intervention effects, and as humane endpoint indicators. Published consensus guidelines have been available for over a decade that specify limits to tumour size consistent with humane use and study validity (e.g. [3]), and animal ethics oversight committees usually have clear specifications for maximum permissible tumour size. It was therefore disappointing that this survey showed that these items were poorly reported.

Evaluation of tumorigenesis data is further confounded by lack of consistent standards for tumour volume determinations. External calliper measurements (the most commonly used method) are prone to major systematic biases and observer variability [18, 79]. These problems are exacerbated by different methods of estimating tumour volume from linear measurements, which may result in considerable under- or over -estimation of tumour sizes. Because certain cancers have the potential for explosive tumour growth, measurement frequency should be tailored to the specific cancer type to avoid tumours exceeding allowable limits between measurement intervals. The inappropriate reporting of ‘relative’ or ‘normalised’ tumour volumes, alternative metrics or methods of calculating volume that do not relate reliably to tumour burden, plus failure to define maximum time and burden limits, also contribute to lack of transparency and oversight.

Methods of euthanasia can induce large differences in protein, metabolite, and biomarker expression, depending on both agent and pre-euthanasia versus post-euthanasia timing of tissue collection [80–82]. Our finding that 80% (321/400) of articles in this survey did not report any verifiable euthanasia methods at all is also concerning, as it is therefore impossible to compare results based on tissue harvest data.

Previous reviews have reported uniformly disappointing results for reporting of study validity and risk of bias items [10, 19, 21, 83]. This survey showed shared the (sadly) common features of inadequate sample size reporting, inappropriate justification, and poor understanding of basic concepts involved with bias minimisation. The extent of claimed randomisation observed in this study is probably greatly exaggerated, as it is likely that many investigators conflate ‘random’ with ‘haphazard’ or ‘unplanned’, and no study provide sufficient detail to indicate if the appropriate units of analysis were used in subsequent hypothesis tests. There was no indication that journal impact factor made much, if any, difference to risk of bias in published articles.

Limitations of this study include the potential for selection bias and lack of quality appraisal of the included studies [7, 84]. We selected only oncology journals from the larger established publishing groups, excluded those published in a language other than English, and included only those that explicitly endorsed the ARRIVE guidelines. Because reporting guidelines represent the minimum information necessary for assessing research reliability [9], we reasoned that journals endorsing such guidelines would provide a compliance benchmark for other journals not following such guidelines. However, this was not the case. Reporting compliance overall was extremely poor, with major methodological reporting gaps and no apparent relationship between rates of reporting and journal impact factor. Second, because this was a cross-sectional survey and not a formal systematic review, we did not evaluate the quality of evidence for individual studies, differences between cancer models, or validity of results [83]. Instead our goal was to determine patterns of reporting and reporting gaps for animal care and welfare items known to contribute to variability in response to both cancer induction methods and experimental interventions. We cannot rule out bias in the studies themselves. Perceived bias against publication of ‘‘negative” results may mean that investigators probably do not report all experiments with all animals, but only those with “significant” findings [83].

A recent survey of preclinical investigators indicated that the major reason for failure to report ARRIVE items in research reports was because those items were not considered ‘important’ or ‘necessary’ [20]. The widespread omissions noted in this survey indicate that animal-related information, although specifically singled out in the ARRIVE guidelines, definitely takes a back seat to that for other resources and procedures. Unfortunately, reporting quality has remained consistently poor across diverse research specialties and journals since the guidelines were introduced [10, 19]. One factor contributing to the lack of improvement is the current publication fashion for the reporting of numerous diverse experiments in a single paper, presumably to indicate if results are ‘robust’. This has resulted in highly information-dense reporting of the results for numerous individual experiments and incomplete methodology documentation, making studies difficult or impossible to review for substance [85]. A second factor is the widespread failure of journal and peer reviewers to actively enforce agreed-upon best-practice reporting standards [19, 20].

Investigators, journal editors, and peer reviewers need to put the ‘mouse’ back into mouse model-based research. Editorial and journal staff must be more actively involved in enforcing reporting standards [9, 20] and ensure that all relevant animal-based information (including details of ethical oversight) are described. Mandatory completion of ARRIVE or Structured, Transparent, Accessible Reporting (STAR Methods) checklists by the submitting authors has been documented to significantly improve scientific reporting when enforced by journals [86–88]. The reform of journal content in the direction of fewer, but better and more thoroughly documented and reported experiments should be prioritised. This would have the added advantage of reducing information overload and therefore the burden on reviewers, and possibly contribute to more thorough reviews [85].

Accountability in science is key to improving practices. It is a scientific imperative to ensure that models are relevant and translatable. It is an ethical imperative to minimize pain and distress in animals used in invasive research and that appropriate oversight guardrails are in place. If methodological substance is de-emphasised in favour of the narrative of results, preclinical oncology research will continue to be compromised.

## Supporting information

S1 Checklist Preferred Reporting Items for Systematic reviews and Meta-Analyses extension for Scoping Reviews (PRISMA-ScR) checklist. (PDF)

S1 File Supplemental methods. (DOCX)

S2 File Supplemental results, S1-S6 Tables. (DOCX)

S1 Data (XLSX)
