# Supplementary material for: ‘Invisible actors’—How poor methodology reporting compromises mouse models of oncology: A cross-sectional survey
Source: PLoS One. 2022 Oct 20;17(10):e0274738. doi: 10.1371/journal.pone.0274738 (PMC9584398; doi:10.1371/journal.pone.0274738)
Supplement: S1 File — (DOCX) [file pone.0274738.s002.docx]

**S1. Supplemental Methods**

### Journal selection

### One author (PR) conducted searches in the National Library of Medicine journal database ((search[oncology OR cancer] [*Journal*] AND (MEDLINE[oncology OR cancer] OR EMBASE[oncology OR cancer]); English language)) on three separate occasions (11 June 2020; 18 September 2020, 8 November, 2020). A total of 284 journals related to oncology research were extracted. Journals were not included if the journal was discontinued, not indexed in either Medline or Embase, Directory of Open Access Journals (DOAJ), and/or Clarivate Web of Science, and not in English. Journals were also excluded if the focus was on clinical or cell-line research, reviews (rather than original research), clinical case reports, or if experiments using whole animals were not described. ‘Instructions to Authors’ and journal reporting expectations for each journal were then examined, and journals excluded if ARRIVE reporting guidelines were not explicitly endorsed. Journal selection was restricted to a maximum of four per publishing group to ensure relatively uniform coverage across publishers (Instructions to Authors guidelines tended to be similar or identical for journals within publishing groups). Each extracted journal was then opened on the journal online content archive page, and beginning with each January 2020 issue, electronic searching on the terms ((mouse OR mice OR murine) OR preclinical OR animal)) and all article titles, abstracts, and main text individually screened for content. The first twenty articles in each journal that explicitly described mouse-based research were selected. Articles describing clinical or epidemiological studies, *in vitro* studies, letters to the editor, conference abstracts, and reviews were excluded. Impact factor of each journal was obtained from Clarivate Web of Science 2020 *InCites Journal Citation* *Reports* <https://jcr.clarivate.com/JCRJournalHomeAction.action> (S1 Table 1).

Checklist of reporting items is given below (**S1 Table 2**). Items were scored as reported (yes=1; no=0). ***Ethical oversight reporting*** consisted of three items: an explicit statement of approval by the relevant institutional ethical committee, a verifiable protocol approval or project license number, and a statement of compliance with verifiable institutional, national, and/or international guidelines or directives for animal care and use. Statements such as “Animal studies were carried out in accordance with the NIH Guide for the Care and Use of Laboratory Animals” were not considered synonymous with institutional approval of the protocol. Numbers provided were also checked against NIH Office of Laboratory Animal Welfare (OLAW*) Institutions with a PHS Approved Animal Welfare Assurance* listings (***https://olaw.nih.gov/assured/app/index.html***) to determine if they referred to a generalised institutional assurance number rather than a specific protocol number. Assurance of adherence to accepted standards of animal care and use had to refer to verifiable guidelines, (e.g. NIH *Guide to the Care and Use of Laboratory Animals*), or a named institution and/or ethical oversight committee. Vague statements such as “compliance with the Declaration of Helsinki”, or non-verifiable statements such as “any applicable institutional guidelines”, or "Animals were maintained according to the stated guidelines of the 3Rs (replacement, reduction, and refinement)" were not scored as reported.

***Animal signalment.*** Items were strain, age, sex, and body weight. Strain identification consisted of both a nominal mouse strain (e.g. C57BL/6, BALB/c, NOD/SCID) plus verifiable strain identifiers such as Research Resource Identifiers (RRID), vendor stock or strain number, and/or detailed information on the genetic crosses used to generate animals. ‘Source’ consisted of recognised vendors, repositories, laboratories, or other verifiable suppliers, or an explicit declaration of in-house colony derivation. Age was identified in units of time (weeks, months) and sex explicitly identified as ‘male’, ‘female’, or both. Statements such as “age and sex-matched animals were used” were not scored as reported because it could not be determined which ages and sexes were used. Animal husbandry items were caging, stocking density (numbers of animals per cage), provision of enrichment, environmental factors (temperature, photoperiod), maintenance food (diet identifier, manufacturer, diet formulation if applicable), feeding (*ad lib/*unrestricted, restricted), and acclimation period prior to experimentation. Welfare items were anesthesia (agent, route, doses/concentrations, method of administration), analgesia (agent, administration method, timing of administration [pre-emptive, post-operative], opioid use), and an explicit specification of humane endpoints. Euthanasia methods were scored as ‘Methods reported’ if one or more methods was explicitly identified, ‘Not specified’ if a euphemism was used (‘killed’, ‘sacrificed’, ‘euthanised’, ‘harvested’, ‘terminated’), and as ‘Not reported’ if no killing method prior to tissue harvest was mentioned.

***Tumour burden.*** Reporting of methods of tumour induction and tumour burden was assessed in a subset of articles describing superficial tumour models (n=290; **S1 Table 3**). Main text, methods, results, figures, and supplementary files were screened for pertinent information. We scored the following items as reported or not reported: anatomical site, site laterality (whether the methods described if tumour inoculant was introduced on one or both sides, unilateral/bilateral), if callipers were used, method for computing volume or volume formula, maximum tumour size, and time to achieve maximum tumour size. Absolute subcutaneous tumour sizes exceeding 1500 mm^3^ were tabulated as 1 = exceeded limits , 0 = did not exceed limits; “not reported”, or “relative” if only a relative, normalised, or nonstandard metric was used.

***Study validity.*** All articles were assessed for reporting of total number of mice used, sample size per group, sample size justification with power calculations, non-statistical justification of animal numbers (e.g. “previous experience”), and bias minimisation methods (randomisation, blinding). Almost no articles in the sample provided sufficient verifiable detail on how methods were performed. Because descriptions were for the most part vague and unreliable, items were scored as reported if key words were used (e.g. “random”, blinding”), but data were not formally analysed other than summarising as counts and percentages.

**Data quality assurance.** Both authors collaborated in the development of the data dictionary and agreed on working definitions for all terms. Both authors scored articles independently and coded entries in separate Excel spreadsheets. Spreadsheets were then combined into the same workbook for cell by cell comparison. Discrepancies were flagged using the Excel formula =IF(PR!A2<>EN!A2,"PR :"&PR!A2&CHAR(10)&" EN:"&EN!A2,""). Flagged entries were checked against the original article. Errors (typos, stray characters, missed information) were corrected, and any further discrepancies resolved by discussion and consensus.

**Exemplary SAS code for hierarchical generalised linear models.** The probability of reporting compliance for each item was estimated using a two-level generalised random-intercept model, with articles clustered within journal, no predictors, and dichotomous outcomes. ‘JOURNAL’ is the level-2 classification variable. ‘Article’ effects are level-1 residuals clustered within JOURNAL. Models were fitted using Laplace estimation in SAS *proc glimmix* (SAS v.9.4, SAS Institute, Cary NC) for each reporting item (designated by Y). Exemplary SAS code was adapted from [11] as follows:

/*Y is the response variable */

/*Article data are level-1 residuals clustered within JOURNAL (level-2)*/

/*Variance components VC are used to obtain the variances for calculating φ and ICC*/

/*CL obtains the 95% confidence limits on the estimates*/

**proc** **glimmix** data=mouse method=laplace;

class JOURNAL;

model Y(event='1') = / solution CL dist=binary link=logit;

random int / subject= JOURNAL type=VC CL;

COVTEST /WALD CL;

run;

The estimate provided in the ‘Solution for Fixed Effects’ table is the log odds of reporting the specified item at a ‘typical’ journal (u_0j_ = 0). See [11] for further details.

**S1 Table 1.** Selected journals and impact factors, obtained from Clarivate Web of Science 2020 Journal Citation Reports

| **Publishing group** | **Journal** | **Impact factor** |
| --- | --- | --- |
| AACR | *Cancer Discovery* | 29.497 |
| AACR | *Cancer Immunology Research* | 8.728 |
| AACR | *Cancer Research* | 9.727 |
| AACR | *Molecular Cancer Research* | 4.630 |
| BMC | *BMC Cancer* | 3.150 |
| BMC | *Cancer Cell International* | 4.175 |
| BMC | *Journal Hematology & Oncology* | 11.059 |
| BMC | *Molecular Cancer* | 15.302 |
| Elsevier | *Cancer Cell* | 26.602 |
| Elsevier | *Cancer Letters* | 7.360 |
| Elsevier | *Neoplasia* | 5.696 |
| Elsevier | *Translational Oncology* | 3.558 |
| Nature | *British Journal of Cancer* | 5.791 |
| Nature | *Cancer Gene Therapy* | 4.534 |
| Nature | *Oncogene* | 7.971 |
| Springer | *Cancer Chemotherapy and Pharmacology* | 2.967 |
| Springer | *Cancer Immunology Immunotherapy* | 5.442 |
| Wiley | *Cancer Medicine* | 3.491 |
| Wiley | *International Journal of Cancer* | 5.145 |
| Wiley | *Molecular Oncology* | 6.574 |

**S1 Table 2: Checklist of animal care and welfare reporting items**

| **QUERY ITEM** | **REPORTED** | |
| --- | --- | --- |
|  | **YES** | **NO** |
| *1 Ethical oversight statement* |  |  |
| Institutional approval explicitly stated |  |  |
| Verifiable protocol institutional license or permit number |  |  |
| Verifiable national or international guidelines for animal care and use referenced |  |  |
| 2*. Animal model* |  |  |
| Official strain designation |  |  |
| RRID, stock number. If in-house, description of strain derivation and QA procedures |  |  |
| Age |  |  |
| Sex |  |  |
| Male |  |  |
| Female |  |  |
| Both |  |  |
| Body weight |  |  |
| Verifiable vendor, source |  |  |
| *3. Animal husbandry* |  |  |
| Acclimation period, if applicable |  |  |
| Caging (type, ventilation, bedding, etc.) |  |  |
| Stocking density (number of animals per cage) |  |  |
| Enrichment provided |  |  |
| Environment |  |  |
| Temperature |  |  |
| Photoperiod |  |  |
| Food and feeding |  |  |
| Diet identified |  |  |
| Amount, frequency (ad lib, restricted) |  |  |
| *4. Welfare* |  |  |
| Anesthesia (use reported: agent, dose, route) |  |  |
| Analgesia (use reported: agent, dose, route) |  |  |
| Humane endpoints explicitly identified |  |  |
| 5*. Euthanasia* |  |  |
| Methods reported |  |  |
| Physical |  |  |
| Inhalation |  |  |
| Injectable drug (agent, dose, route) |  |  |
| Not specified; euphemism used (“sacrificed”, “euthanised”, etc.) |  |  |
| Not reported |  |  |

**S1 Table 3:** Checklist of reporting items for tumour induction and tumour burden

| **QUERY ITEM** | **REPORTED** | |
| --- | --- | --- |
|  | **YES** | **NO** |
| *1 Induction* |  |  |
| Anatomical site |  |  |
| Laterality: Unilateral or bilateral |  |  |
| Route (subcutaneous, spontaneous, intraperitoneal, orthotopic, etc) |  |  |
| 2*. Tumour metrics* |  |  |
| Method of measurement (e.g. calipers) |  |  |
| Volume calculation formula |  |  |
| Frequency of measurement |  |  |
| Maximum tumour size |  |  |
| Time from tumour induction to euthanasia or experiment endpoint |  |  |
| Tumour volume exceeds 1500 mm^3^ |  |  |
|  | Not reported | |
|  | Relative, ‘normalised’ | |

**S1 Table 4:** Checklist of reporting study validity items

| **QUERY ITEM** | **REPORTED** | |
| --- | --- | --- |
|  | **YES** | **NO** |
| Total number of animals used |  |  |
| Sample size justification using power calculations |  |  |
| Other justifications for sample size (e.g. “previous experience”, etc.) |  |  |
| Sample size per group |  |  |
| Randomisation performed |  |  |
| Concealment/blinding (allocation, operator, assessors) |  |  |
