# Supplementary material for: ‘Invisible actors’—How poor methodology reporting compromises mouse models of oncology: A cross-sectional survey
Source: PLoS One. 2022 Oct 20;17(10):e0274738. doi: 10.1371/journal.pone.0274738 (PMC9584398; doi:10.1371/journal.pone.0274738)
Supplement: S2 File — (DOCX) [file pone.0274738.s003.docx]

**S2. Supplemental Results**

Compliance reporting for 22 animal-related items in five domains for 20 oncology journals, by journal.

**S2 Table 1.** **Ethical oversight reporting**

| **Journal** | **Institutional approval** | **Approval number** | **Guidelines** | **No statement** |
| --- | --- | --- | --- | --- |
| *Cancer Discovery* | 13 | 0 | 9 | 1 |
| *Cancer Immunol Res* | 19 | 6 | 7 | 0 |
| *Cancer Research* | 18 | 7 | 9 | 1 |
| *Molecular Cancer Research* | 18 | 6 | 4 | 1 |
| *BMC Cancer* | 17 | 9 | 12 | 1 |
| *Cancer Cell International* | 15 | 0 | 5 | 1 |
| *Journal Hematology & Oncology* | 16 | 3 | 7 | 2 |
| *Molecular Cancer* | 15 | 4 | 8 | 3 |
| *Cancer Cell* | 19 | 8 | 7 | 0 |
| *Cancer Letters* | 17 | 4 | 10 | 1 |
| *Neoplasia* | 16 | 2 | 5 | 4 |
| *Transl Oncol.* | 15 | 7 | 13 | 1 |
| *Br J Cancer* | 19 | 8 | 14 | 0 |
| *Cancer Gene therapy* | 19 | 5 | 10 | 1 |
| *Oncogene* | 17 | 3 | 8 | 0 |
| *Cancer Chemother Pharmacol.* | 14 | 6 | 13 | 1 |
| *Cancer Immunol Immunother* | 20 | 16 | 15 | 0 |
| *Cancer Medicine* | 17 | 2 | 2 | 3 |
| *International Journal of Cancer* | 12 | 4 | 8 | 4 |
| *Molecular Oncology* | 15 | 5 | 8 | 2 |
| **Counts per item, n** | 331 | 105 | 174 | 27 |
| **Percent (total N = 400)** | ***83*** | ***26*** | ***44*** | ***7*** |

**S2 Table 2.** Animal strain and source reporting

| **Journal** | **Strain:**  **Nominal identifiers** | **Strain:**  **Verifiable identifiers** | **Vendor/**  **Source** |
| --- | --- | --- | --- |
| *Cancer Discovery* | 19 | 3 | 15 |
| *Cancer Immunol Res* | 20 | 2 | 19 |
| *Cancer Research* | 20 | 3 | 14 |
| *Molecular Cancer Research* | 15 | 3 | 15 |
| *BMC Cancer* | 18 | 1 | 19 |
| *Cancer Cell International* | 18 | 0 | 16 |
| *Journal Hematology & Oncology* | 18 | 0 | 12 |
| *Molecular Cancer* | 15 | 0 | 9 |
| *Cancer Cell* | 20 | 12 | 19 |
| *Cancer Letters* | 16 | 0 | 15 |
| *Neoplasia* | 20 | 3 | 11 |
| *Transl Oncol.* | 14 | 1 | 12 |
| *Br J Cancer* | 19 | 1 | 17 |
| *Cancer Gene therapy* | 18 | 0 | 15 |
| *Oncogene* | 16 | 0 | 13 |
| *Cancer Chemother Pharmacol.* | 19 | 0 | 13 |
| *Cancer Immunol Immunother* | 20 | 1 | 20 |
| *Cancer Medicine* | 15 | 3 | 14 |
| *International Journal of Cancer* | 18 | 1 | 11 |
| *Molecular Oncology* | 18 | 0 | 1 |
|  |  |  |  |
| **Counts per item, n** | 356 | 34 | 280 |
| **Percent (total N = 400)** | ***89*** | ***9*** | ***70*** |

**S2 Table 3. Animal signalment**

| **Journal** | **Age** | **Sex** | **Weight** |
| --- | --- | --- | --- |
| *Cancer Discovery* | 19 | 11 | 0 |
| *Cancer Immunol Res* | 19 | 13 | 0 |
| *Cancer Research* | 13 | 11 | 0 |
| *Molecular Cancer Research* | 15 | 14 | 1 |
| *BMC Cancer* | 19 | 16 | 8 |
| *Cancer Cell International* | 18 | 16 | 2 |
| *Journal Hematology & Oncology* | 14 | 13 | 0 |
| *Molecular Cancer* | 16 | 11 | 2 |
| *Cancer Cell* | 17 | 17 | 1 |
| *Cancer Letters* | 18 | 18 | 5 |
| *Neoplasia* | 15 | 12 | 0 |
| *Transl Oncol.* | 15 | 13 | 2 |
| *Br J Cancer* | 18 | 19 | 6 |
| *Cancer Gene therapy* | 16 | 16 | 5 |
| *Oncogene* | 17 | 19 | 0 |
| *Cancer Chemother Pharmacol.* | 14 | 13 | 3 |
| *Cancer Immunol Immunother* | 16 | 16 | 3 |
| *Cancer Medicine* | 16 | 12 | 2 |
| *International Journal of Cancer* | 16 | 14 | 1 |
| *Molecular Oncology* | 14 | 13 | 0 |
| Counts per item, n | 325 | 287 | 41 |
| Percent (total N = 400) | ***81*** | ***72*** | ***10*** |

**S2 Table 4. Animal housing and husbandry**

| **Journal** | **Caging** | **Density** | **Enrichment** |
| --- | --- | --- | --- |
| *Cancer Discovery* | 1 | 1 | 0 |
| *Cancer Immunol Res* | 0 | 1 | 0 |
| *Cancer Research* | 0 | 0 | 0 |
| *Molecular Cancer Research* | 0 | 0 | 0 |
| *BMC Cancer* | 3 | 5 | 1 |
| *Cancer Cell International* | 0 | 0 | 0 |
| *Journal Hematology & Oncology* | 1 | 0 | 0 |
| *Molecular Cancer* | 0 | 0 | 0 |
| *Cancer Cell* | 3 | 1 | 0 |
| *Cancer Letters* | 0 | 0 | 0 |
| *Neoplasia* | 1 | 1 | 0 |
| *Transl Oncol.* | 0 | 0 | 0 |
| *Br J Cancer* | 3 | 3 | 3 |
| *Cancer Gene therapy* | 2 | 1 | 0 |
| *Oncogene* | 0 | 0 | 0 |
| *Cancer Chemother Pharmacol.* | 1 | 0 | 0 |
| *Cancer Immunol Immunother* | 0 | 1 | 0 |
| *Cancer Medicine* | 1 | 0 | 0 |
| *International Journal of Cancer* | 0 | 0 | 0 |
| *Molecular Oncology* | 0 | 0 | 0 |
| Counts per item, n | 16 | 14 | 4 |
| ***Percent (total N = 400)*** | ***4*** | ***4*** | ***1*** |

**S2 Table 4. Animal housing and husbandry (cont.)**

| **Journal** | **Temperature** | **Photoperiod** | **Food and water** | **Feed** | **Acclimation** |
| --- | --- | --- | --- | --- | --- |
| *Cancer Discovery* | 0 | 1 | 4 | 5 | 1 |
| *Cancer Immunol Res* | 0 | 0 | 1 | 2 | 1 |
| *Cancer Research* | 1 | 2 | 1 | 0 | 1 |
| *Molecular Cancer Research* | 0 | 0 | 1 | 1 | 0 |
| *BMC Cancer* | 6 | 7 | 5 | 1 | 2 |
| *Cancer Cell International* | 3 | 3 | 5 | 0 | 0 |
| *Journal Hematology & Oncology* | 0 | 0 | 0 | 0 | 1 |
| *Molecular Cancer* | 0 | 0 | 0 | 0 | 1 |
| *Cancer Cell* | 1 | 3 | 4 | 0 | 2 |
| *Cancer Letters* | 2 | 4 | 2 | 0 | 1 |
| *Neoplasia* | 0 | 1 | 2 | 2 | 0 |
| *Transl Oncol.* | 3 | 4 | 2 | 1 | 0 |
| *Br J Cancer* | 7 | 11 | 9 | 3 | 0 |
| *Cancer Gene therapy* | 1 | 3 | 2 | 0 | 2 |
| *Oncogene* | 0 | 1 | 2 | 0 | 1 |
| *Cancer Chemother Pharmacol.* | 3 | 3 | 4 | 1 | 3 |
| *Cancer Immunol Immunother* | 0 | 1 | 1 | 0 | 0 |
| *Cancer Medicine* | 1 | 1 | 1 | 0 | 1 |
| *International Journal of Cancer* | 0 | 0 | 0 | 0 | 0 |
| *Molecular Oncology* | 0 | 0 | 1 | 0 | 0 |
| Counts per item, n | 28 | 45 | 47 | 16 | 17 |
| Percent (total N = 400) | 7 | 11 | 12 | 4 | 4 |

**S2 Table 5. Welfare-related reporting**

| **Journal** | **Anesthesia** | **Analgesia** | **Humane endpoints** |
| --- | --- | --- | --- |
| *Cancer Discovery* | 4 | 1 | 3 |
| *Cancer Immunol Res* | 3 | 2 | 6 |
| *Cancer Research* | 3 | 1 | 0 |
| *Molecular Cancer Research* | 4 | 0 | 4 |
| *BMC Cancer* | 6 | 0 | 3 |
| *Cancer Cell International* | 1 | 0 | 3 |
| *Journal Hematology & Oncology* | 0 | 0 | 4 |
| *Molecular Cancer* | 3 | 0 | 1 |
| *Cancer Cell* | 6 | 0 | 4 |
| *Cancer Letters* | 0 | 0 | 3 |
| *Neoplasia* | 3 | 0 | 4 |
| *Transl Oncol.* | 5 | 1 | 4 |
| *Br J Cancer* | 10 | 3 | 4 |
| *Cancer Gene therapy* | 1 | 0 | 6 |
| *Oncogene* | 2 | 1 | 3 |
| *Cancer Chemother Pharmacol.* | 2 | 1 | 2 |
| *Cancer Immunol Immunother* | 2 | 1 | 5 |
| *Cancer Medicine* | 2 | 0 | 1 |
| *International Journal of Cancer* | 0 | 0 | 4 |
| *Molecular Oncology* | 2 | 0 | 0 |
| Counts per item, n | **59** | **11** | **64** |
| Percent (total N = 400) | ***15*** | ***3*** | ***16*** |

**S2 Table 6. Euthanasia reporting**

|  | **Euthanasia method** | | |
| --- | --- | --- | --- |
| **Journal** | **Identified** | **Not specified** | **Not reported** |
| *Cancer Discovery* | 2 | 9 | 9 |
| *Cancer Immunol Res* | 3 | 14 | 3 |
| *Cancer Research* | 1 | 7 | 11 |
| *Molecular Cancer Research* | 3 | 9 | 8 |
| *BMC Cancer* | 18 | 0 | 2 |
| *Cancer Cell International* | 1 | 14 | 5 |
| *Journal Hematology & Oncology* | 0 | 14 | 6 |
| *Molecular Cancer* | 1 | 15 | 4 |
| *Cancer Cell* | 2 | 10 | 8 |
| *Cancer Letters* | 1 | 14 | 5 |
| *Neoplasia* | 3 | 13 | 4 |
| *Transl Oncol.* | 7 | 9 | 4 |
| *Br J Cancer* | 18 | 2 | 0 |
| *Cancer Gene therapy* | 1 | 12 | 7 |
| *Oncogene* | 4 | 11 | 5 |
| *Cancer Chemother Pharmacol.* | 5 | 7 | 8 |
| *Cancer Immunol Immunother* | 2 | 14 | 4 |
| *Cancer Medicine* | 3 | 15 | 2 |
| *International Journal of Cancer* | 2 | 11 | 7 |
| *Molecular Oncology* | 1 | 13 | 6 |
| Counts per item, n | **78** | **213** | **108** |
| Percent (total N = 400) | ***20*** | ***53*** | ***27*** |

**S2 Table 7. Study validity item reporting.**

Sample size: Total= total numbers of animal used; Group= sample size per group or arm;

Sample size justification: Power = formal power calculations; Other= any rationale given for numbers used;

Bias minimisation: Random = Randomisation used to allocate interventions to experimental units; Conceal: Blinding or concealment of allocation to any of operators or assessors.

|  | **Sample size** | | **Sample size justification** | | **Bias minimisation** | |
| --- | --- | --- | --- | --- | --- | --- |
| **Journal** | **Total** | **Group** | **Power** | **Other** | **Random** | **Conceal** |
| *Cancer Discovery* | 0 | 2 | 0 | 0 | 4 | 0 |
| *Cancer Immunol Res* | 0 | 3 | 0 | 0 | 7 | 2 |
| *Cancer Research* | 0 | 1 | 0 | 1 | 7 | 0 |
| *Molecular Cancer Research* | 5 | 5 | 0 | 0 | 6 | 1 |
| *BMC Cancer* | 2 | 7 | 0 | 1 | 9 | 0 |
| *Cancer Cell International* | 0 | 3 | 0 | 0 | 10 | 2 |
| *Journal Hematology & Oncology* | 10 | 9 | 2 | 2 | 10 | 0 |
| *Molecular Cancer* | 4 | 7 | 0 | 0 | 6 | 0 |
| *Cancer Cell* | 3 | 2 | 1 | 4 | 13 | 2 |
| *Cancer Letters* | 4 | 4 | 0 | 0 | 6 | 0 |
| *Neoplasia* | 6 | 12 | 0 | 0 | 8 | 2 |
| *Transl Oncol.* | 2 | 6 | 0 | 0 | 8 | 1 |
| *Br J Cancer* | 6 | 11 | 0 | 0 | 8 | 0 |
| *Cancer Gene therapy* | 1 | 5 | 0 | 0 | 7 | 0 |
| *Oncogene* | 3 | 13 | 0 | 2 | 13 | 1 |
| *Cancer Chemother Pharmacol.* | 2 | 9 | 1 | 4 | 13 | 3 |
| *Cancer Immunol Immunother* | 3 | 7 | 1 | 0 | 12 | 0 |
| *Cancer Medicine* | 1 | 5 | 1 | 0 | 6 | 2 |
| *International Journal of Cancer* | 3 | 3 | 1 | 0 | 6 | 0 |
| *Molecular Oncology* | 4 | 10 | 0 | 2 | 6 | 0 |
| Counts per item, n | **59** | **124** | **7** | **16** | **165** | **16** |
| Percent (total N = 400) | ***15*** | ***31*** | ***2*** | ***4*** | ***41*** | ***4*** |
